# Analysis of anemia and iron supplementation among glioblastoma patients reveals sex-biased association between anemia and survival

**DOI:** 10.1038/s41598-024-52492-8

**Published:** 2024-01-29

**Authors:** Ganesh Shenoy, Becky Slagle-Webb, Chachrit Khunsriraksakul, Bhavyata Pandya Shesh, Jingqin Luo, Vladimir Khristov, Nataliya Smith, Alireza Mansouri, Brad E. Zacharia, Sheldon Holder, Justin D. Lathia, Jill S. Barnholtz-Sloan, James R. Connor

**Affiliations:** 1 Department of Neurosurgery, Penn State College of Medicine, Hershey, PA USA; 2 Department of Public Health Sciences, Penn State College of Medicine, Hershey, PA USA; 3 Division of Public Health Sciences, Department of Surgery and Siteman Cancer Center Biostatistics Shared Resource, Washington University School of Medicine, St. Louis, MO USA; 4 Department of Pathology and Laboratory Medicine, Brown University, Providence, RI USA; 5 Lerner Research Institute, Cleveland Clinic, Cleveland, OH USA; 6 Trans-Divisional Research Program, Division of Cancer Epidemiology and Genetics, National Cancer Institute, Bethesda, MD USA; 7 Center for Biomedical Informatics and Information Technology, National Cancer Institute, Bethesda, MD USA

**Keywords:** CNS cancer, CNS cancer

## Abstract

The association between anemia and outcomes in glioblastoma patients is unclear. We analyzed data from 1346 histologically confirmed adult glioblastoma patients in the TriNetX Research Network. Median hemoglobin and hematocrit levels were quantified for 6 months following diagnosis and used to classify patients as anemic or non-anemic. Associations of anemia and iron supplementation of anemic patients with median overall survival (median-OS) were then studied. Among 1346 glioblastoma patients, 35.9% of male and 40.5% of female patients were classified as anemic using hemoglobin-based WHO guidelines. Among males, anemia was associated with reduced median-OS compared to matched non-anemic males using hemoglobin (HR 1.24; 95% CI 1.00–1.53) or hematocrit-based cutoffs (HR 1.28; 95% CI 1.03–1.59). Among females, anemia was not associated with median-OS using hemoglobin (HR 1.00; 95% CI 0.78–1.27) or hematocrit-based cutoffs (HR: 1.10; 95% CI 0.85–1.41). Iron supplementation of anemic females trended toward increased median-OS (HR 0.61; 95% CI 0.32–1.19) although failing to reach statistical significance whereas no significant association was found in anemic males (HR 0.85; 95% CI 0.41–1.75). Functional transferrin-binding assays confirmed sexually dimorphic binding in resected patient samples indicating underlying differences in iron biology. Anemia among glioblastoma patients exhibits a sex-specific association with survival.

## Introduction

Glioblastoma remains one of the most difficult-to-treat malignancies facing modern oncology. Despite maximal treatment with surgical resection, chemotherapy, and radiation, patients face poor prognosis with 5-year survival rates of less than 10%^1^. Given these poor outcomes, more research is urgently needed to better understand the underlying biological processes driving glioblastoma tumor progression and poor patient outcomes.

Anemia is a common yet undertreated condition with global prevalence estimates as high as 22.8% and even higher prevalence in older individuals, females, and those with chronic diseases such as cancer^2^. The underlying cause of anemia varies by country and degree of development, but iron deficiency remains the most common cause of anemia globally, including in developed countries^2^. Among cancer patients, functional iron deficiency anemia is the most common cause and thought to be brought about by the inflammatory nature of the tumor as well as through the side effects of cancer treatment^3^.

The biological effects of anemia and its downstream consequences on human pathophysiology are closely intertwined with oxygen delivery to tumor and surrounding tissues. It has been reported that anemia is directly associated with reduced tumor oxygenation in several different types of cancers. In breast cancer, for example, Vaupel et al.^4^ found that hemoglobin concentration linearly correlated with tumor oxygenation. Reduction of oxygen delivery to tumor and tissues is known to stabilize hypoxia-inducible factors which subsequently act as transcription factors leading to a wide variety of phenotypic changes^5^. Several specific examples of the consequences of hypoxic signaling include: increased angiogenesis via enhanced vascular endothelial growth factor (VEGF) secretion^6^, promotion of an immunosuppressive microenvironment by reprogramming of tumor-associated macrophages^7^, increased activation of myeloid-derived suppressor cells^8^, and inhibition of T-cell mediated ant-tumor immunity^9,10^. Anemia-induced hypoxia may also contribute to treatment resistance given the necessity of molecular oxygen for optimal radiotherapy^11^. Upregulation of drug transporters and inhibition of apoptotic pathways that contribute to treatment resistance has also been reported to be associated with tumor hypxoia^12^. Unsurprisingly, anemia has been unfavorably associated with the progression of numerous types of malignancies including breast^13^, colorectal^14,15^, lung^16^, and urologic cancers^17^.

Although the existing literature make a compelling argument as to why correcting anemia could be favorable, the field of tumor iron biology has put forward arguments against providing iron to tumors. Iron is a critical factor for functions that enhance cancer cell growth and tumorigenesis such as DNA synthesis, increased oxidative metabolism and induced autophagy^18–20^. Transcriptomic profiling has also suggested that profiles supporting iron acquisition may be associated with more aggressive phenotypes^21^. Nonetheless, recent work has also highlighted novel aspects of iron biology that may support an anti-tumor role including the promotion of cancer cell ferroptosis^22^, inhibition of glioblastoma cell migration^23^, and promotion of anti-tumor immunity^24^.

The conflicting arguments between correcting anemia and iron supplementation described above may explain why there are few studies examining anemia, iron supplementation, and outcomes among glioblastoma patients. And given the large disparity between males and females in anemia status in general, clarifying the role of anemia and iron supplementation of anemic patients could provide actionable approaches to improving outcomes in a sex-specific manner. Here we provide the largest analysis of the association between anemia, iron supplementation of anemic patients, and sex-specific glioblastoma survival outcomes to date.

## Methods

### Database and patient cohort

The TriNetX Research Network is a large, federated database containing de-identified data derived from the electronic health records of patients treated in more than 50 healthcare organizations (HCOs) in the United States. The records of 1346 histologically confirmed glioblastoma patients diagnosed between 2005 and 2020 were downloaded from the TriNetX Research Network and used for the analysis reported here. Criteria for inclusion in the analysis were: 18 years of age or older at time of diagnosis, no previous diagnosis of glioblastoma, underwent surgery and had record of histologically confirmed glioblastoma, received at least one hemoglobin, hematocrit, mean corpuscular volume, and white blood cell count lab results on the day of, or within 6 months following glioblastoma diagnosis.

### Classification of anemia and quantification of comorbidities and therapeutics

Logical Observation Identifiers, Names and Codes (LOINC) or TriNetX (TNX) codes were used to identify hemoglobin, hematocrit, mean corpuscular volume, and white blood cell count lab values in each patient’s record from the day of diagnosis to 6 months afterward (Supplementary Table 8). Median hemoglobin and hematocrit levels were calculated for each patient for this time period and used to classify patients as anemic or non-anemic. World Health Organization hemoglobin cutoffs for anemia of 13 g/dL for male and 12 g/dL for female patients were used^25^. Hematocrit cutoffs of 39% for male and 36% for female patients were used as alternate criteria for anemic classification. Comorbidities before glioblastoma diagnosis were identified using ICD-9 and ICD-10 codes (Supplementary Table 6) in the patient record, and a Charlson Comorbidity Index for each patient was calculated using the comorbidity prevalence as described previously^26^. Patients were identified as having received chemotherapy using RxNorm codes for temozolomide, carmustine, or lomustine. Anemic patients were identified as having received an iron supplement using RxNorm codes for a panel of iron compounds (Supplementary Table 7). Patients were considered to have been iron supplemented if the iron supplement was received within 5 years before or 6 months after diagnosis.

### Statistical analysis

All analysis was performed using R version 3.6.3. For the analysis of anemic status and association with median-OS by sex, to account for potential confounders, anemic versus non-anemic cohorts were balanced for the covariates of age at diagnosis, Charlson comorbidity index, whether patients received chemotherapy and/or radiation, time interval from diagnosis to initiation of chemotherapy and/or radiation, white blood cell counts, number of hemoglobin measurements (to account for possible differences in care received between those with a few measurements versus those with much higher measurements), and comorbidities using 1:1 propensity score matching with logistic-regression-based distance calculation and nearest-neighbor matching without replacement and with a caliper distance of 0.1 standard deviation of the logit propensity score using the *MatchIt* package in R^27^. Balance was assessed by examining the absolute standardized mean differences between the covariates of the anemic versus non-anemic cohorts using the *cobalt* package in R (Supplementary Fig. 1A–D)^28,29^. Balance was also assessed by statistical testing of the covariates before and after matching (Tables 1, 2, Supplementary Tables S1, S2). For the analysis of iron supplementation of anemic patients by sex, because of limited sample size, cohorts were balanced only for age at diagnosis, Charlson comorbidity index, and whether patients received chemotherapy and/or radiation (Supplementary Fig. 1E,F). To account for immortal time bias in iron supplemented patients, follow up for patients in the iron supplementation group was adjusted to the date of prescription if the date of first prescription was subsequent to the date of glioblastoma diagnosis as suggested by Zhou et al.^30^. Survival by sex was assessed using the *survival* and *survminer* packages in R with log-rank tests and Cox regression with robust variance estimation to evaluate differences in survival^31–33^.

**Table 1:** Patient characteristics anemic versus non-anemic female patients as defined using hemoglobin cutoffs of 12 g/dL.

Number of patients (N)	Before matching			After matching		
	Hb ≥ 12 g/dL	Hb < 12 g/dL	*P*	Hb ≥ 12 g/dL	Hb < 12 g/dL	*P*
	336	229		187	187	
Continuous variables						
Age at diagnosis	62.53 (11.87)	63.86 (11.66)	0.19	63.63 (11.83)	63.11 (11.78)	0.669
Charlson comorbidity index	0.77 (2.16)	1.19 (2.71)	**0.041**	0.97 (2.21)	1.03 (2.57)	0.829
Median hemoglobin (g/dL)	13.06 (0.73)	10.72 (0.99)	**< 0.001**	13.03 (0.73)	10.84 (0.91)	**< 0.001**
Median hematocrit (%)	39.06 (2.26)	32.67 (3.04)	**< 0.001**	39.03 (2.35)	33.04 (2.81)	**< 0.001**
Median MCV	91.14 (4.29)	89.83 (6.71)	**0.005**	91.44 (4.28)	89.68 (6.58)	**0.002**
Median white blood cell count	9.10 (3.72)	9.06 (4.00)	0.907	9.49 (3.82)	9.10 (3.89)	0.317
Number hemoglobin measurements	14.57 (9.58)	22.08 (19.19)	**< 0.001**	15.81 (10.68)	16.81 (12.36)	0.406
Treatment status						
Received chemotherapy	240 (71.4)	135 (59.0)	**0.003**	114 (61.0)	114 (61.0)	1
Time diagnosis to start of chemotherapy:			**0.014**			0.739
≤ 4 weeks post-diagnosis	159 (47.3)	87 (38.0)	–	74 (39.6)	74 (39.6)	–
4–8 weeks post-diagnosis	43 (12.8)	30 (13.1)	–	19 (10.2)	24 (12.8)	–
> 8 weeks post-diagnosis	38 (11.3)	18 (7.9)	–	21 (11.2)	16 (8.6)	–
Received radiation	188 (56.0)	104 (45.4)	**0.018**	79 (42.2)	88 (47.1)	0.405
Time diagnosis to start of radiotherapy:			**0.045**			0.623
≤ 4 weeks post-diagnosis	67 (19.9)	29 (12.7)	–	17 (9.1)	23 (12.3)	–
4–8 weeks post-diagnosis	100 (29.8)	64 (27.9)	–	54 (28.9)	54 (28.9)	–
> 8 weeks post-diagnosis	21 (6.2)	11 (4.8)	–	8 (4.3)	11 (5.9)	–
Substance use						
Smoking	120 (35.7)	88 (38.4)	0.57	63 (33.7)	65 (34.8)	0.913
Alcohol abuse	12 (3.6)	8 (3.5)	1	7 (3.7)	8 (4.3)	1
Systemic comorbidities						
Myocardial infarction	4 (1.2)	5 (2.2)	0.56	4 (2.1)	2 (1.1)	0.681
Congestive heart failure	6 (1.8)	7 (3.1)	0.482	3 (1.6)	5 (2.7)	0.721
Peripheral vascular disease	8 (2.4)	10 (4.4)	0.282	4 (2.1)	5 (2.7)	1
Cerebrovascular disease	24 (7.1)	28 (12.2)	0.057	20 (10.7)	20 (10.7)	1
Dementia	3 (0.9)	2 (0.9)	1	3 (1.6)	2 (1.1)	1
Chronic pulmonary disease	19 (5.7)	22 (9.6)	0.107	13 (7.0)	13 (7.0)	1
Rheumatic disease	10 (3.0)	10 (4.4)	0.518	6 (3.2)	7 (3.7)	1
Peptic ulcer disease	2 (0.6)	2 (0.9)	1	1 (0.5)	2 (1.1)	1
Mild liver disease	8 (2.4)	8 (3.5)	0.6	5 (2.7)	5 (2.7)	1
Diabetes without complication	23 (6.8)	20 (8.7)	0.503	12 (6.4)	15 (8.0)	0.689
Diabetes with complication	4 (1.2)	2 (0.9)	1	1 (0.5)	2 (1.1)	1
Hemiplegia or paraplegia	6 (1.8)	6 (2.6)	0.705	2 (1.1)	4 (2.1)	0.681
Renal disease	7 (2.1)	8 (3.5)	0.449	6 (3.2)	6 (3.2)	1
Malignancy	18 (5.4)	20 (8.7)	0.161	16 (8.6)	14 (7.5)	0.849
Moderate or severe liver disease	0 (0.0)	0 (0.0)	–	0 (0.0)	0 (0.0)	–
Metastatic cancer	11 (3.3)	14 (6.1)	0.161	9 (4.8)	10 (5.3)	1
HIV or AIDS	3 (0.9)	1 (0.4)	0.901	1 (0.5)	1 (0.5)	1

Columns on the left represent characteristics of the cohorts before propensity score matching and columns on the right represent characteristics of the cohorts after propensity score matching.

Comparisons that were statistically significant using a threshold of *P* < 0.05 are in bold.

**Table 2:** Patient characteristics anemic versus non-anemic male patients as defined using hemoglobin cutoffs of 13 g/dL.

Number of patients (N)	Before matching			After matching		
	Hb ≥ 13 g/dL	Hb < 13 g/dL	*P*	Hb ≥ 13 g/dL	Hb < 13 g/dL	*P*
	501	280		229	229	
Continuous variables						
Age at diagnosis	61.03 (12.51)	64.83 (12.36)	**< 0.001**	64.75 (11.66)	64.18 (12.39)	0.61
Charlson comorbidity index	0.69 (1.70)	1.35 (2.96)	**< 0.001**	1.03 (2.10)	0.91 (2.46)	0.568
Median hemoglobin (g/dl)	14.23 (0.81)	11.71 (1.04)	**< 0.001**	14.12 (0.76)	11.82 (0.99)	**< 0.001**
Median hematocrit (%)	41.95 (2.44)	35.32 (3.15)	**< 0.001**	41.58 (2.34)	35.58 (3.04)	**< 0.001**
Median mcv	90.95 (4.33)	90.50 (6.25)	0.237	91.06 (4.20)	90.47 (6.09)	0.234
Median white blood cell count	9.65 (4.36)	9.45 (3.72)	0.519	9.45 (3.52)	9.55 (3.76)	0.765
Number hemoglobin measurements	15.55 (10.62)	19.76 (16.56)	**< 0.001**	16.85 (12.19)	17.34 (13.60)	0.685
Treatment status						
Received chemotherapy	373 (74.5)	166 (59.3)	**< 0.001**	149 (65.1)	146 (63.8)	0.845
Time diagnosis to start of chemotherapy:			**< 0.001**			0.986
≤ 4 weeks post-diagnosis	254 (50.7)	103 (36.8)	–	96 (41.9)	93 (40.6)	–
4–8 weeks post-diagnosis	71 (14.2)	35 (12.5)	–	30 (13.1)	31 (13.5)	–
> 8 weeks post-diagnosis	48 (9.6)	28 (10.0)	–	23 (10.0)	22 (9.6)	–
Received radiation	274 (54.7)	134 (47.9)	0.079	111 (48.5)	115 (50.2)	0.779
Time diagnosis to start of radiotherapy:			0.245			0.93
≤ 4 weeks post-diagnosis	74 (14.8)	32 (11.4)	–	24 (10.5)	25 (10.9)	–
4–8 weeks post-diagnosis	163 (32.5)	86 (30.7)	–	76 (33.2)	76 (33.2)	–
> 8 weeks post-diagnosis	37 (7.4)	16 (5.7)	–	11 (4.8)	14 (6.1)	–
Substance use						
Smoking	254 (50.7)	141 (50.4)	0.987	115 (50.2)	115 (50.2)	1
Alcohol abuse	48 (9.6)	29 (10.4)	0.823	23 (10.0)	25 (10.9)	0.879
Systemic comorbidities						
Myocardial infarction	12 (2.4)	12 (4.3)	0.211	7 (3.1)	5 (2.2)	0.77
Congestive heart failure	10 (2.0)	13 (4.6)	0.06	7 (3.1)	6 (2.6)	1
Peripheral vascular disease	12 (2.4)	16 (5.7)	0.028	6 (2.6)	5 (2.2)	1
Cerebrovascular disease	40 (8.0)	25 (8.9)	0.747	21 (9.2)	16 (7.0)	0.493
Dementia	2 (0.4)	2 (0.7)	0.945	0 (0.0)	0 (0.0)	–
Chronic pulmonary disease	26 (5.2)	22 (7.9)	0.182	15 (6.6)	13 (5.7)	0.845
Rheumatic disease	5 (1.0)	1 (0.4)	0.578	2 (0.9)	1 (0.4)	1
Peptic ulcer disease	7 (1.4)	3 (1.1)	0.955	3 (1.3)	2 (0.9)	1
Mild liver disease	15 (3.0)	10 (3.6)	0.82	11 (4.8)	7 (3.1)	0.471
Diabetes without complication	23 (4.6)	32 (11.4)	**0.001**	18 (7.9)	18 (7.9)	1
Diabetes with complication	2 (0.4)	9 (3.2)	**0.004**	2 (0.9)	2 (0.9)	1
Hemiplegia or paraplegia	9 (1.8)	9 (3.2)	0.309	5 (2.2)	6 (2.6)	1
Renal disease	6 (1.2)	17 (6.1)	** < 0.001**	6 (2.6)	5 (2.2)	1
Malignancy	32 (6.4)	31 (11.1)	**0.03**	25 (10.9)	20 (8.7)	0.53
Moderate or severe liver disease	1 (0.2)	3 (1.1)	–	1 (0.4)	3 (1.3)	–
Metastatic cancer	14 (2.8)	17 (6.1)	**0.04**	11 (4.8)	10 (4.4)	1
HIV or AIDS	3 (0.6)	1 (0.4)	1	1 (0.4)	1 (0.4)	1

Columns on the left represent characteristics of the cohorts before propensity score matching and columns on the right represent characteristics of the cohorts after propensity score matching.

Comparisons that were statistically significant using a threshold of *P* < 0.05 are in bold.

### Transferrin binding assay

Resected human glioblastoma samples from six male and six female patients were utilized for transferrin binding assays adapted from previously described protocols^34^. The samples were retrieved from the Penn State Hershey Neuroscience Institute Biorepository under an approved institutional review board protocol (IRB #2914). The samples were minced and homogenized in 0.3 M sucrose (VWR Chemicals). Homogenates were centrifuged at 800 × g for 20 min to remove large debris. Supernatants were collected and further centrifuged at 120,000 × g for 1 h and resulting pellets were re-suspended in potassium phosphate buffer supplemented with 10% glycerol (Fisher-Scientific) to isolate plasma membrane extracts. ^125^I-tagged human transferrin (Sigma-Aldrich) was subsequently added to 20 µg of plasma membrane extract in a 96-well filter plate (Millipore-Sigma) and allowed to incubate for 1 h at room temperature. The reaction was then terminated by addition of ice-cold PBS and the mixture was subsequently vacuum filtered using 0.2 µm hydrophilic membranes. Filters were then washed three times with ice-cold PBS to remove unbound radiolabeled transferrin. Radioactivity in the filters was then quantified using a Beckman Gamma 4000 Analyzer.

### Ethics approval and consent to participate

Patient samples utilized in this study were retrieved following a Penn State IRB approved protocol with informed patient consent and fully de-identified prior to distribution to the research team. All clinical data used in this manuscript were obtained fully de-identified. This research was conducted in accordance with the Declaration of Helsinki.

## Results

### Baseline patient characteristics

**Figure 1:**
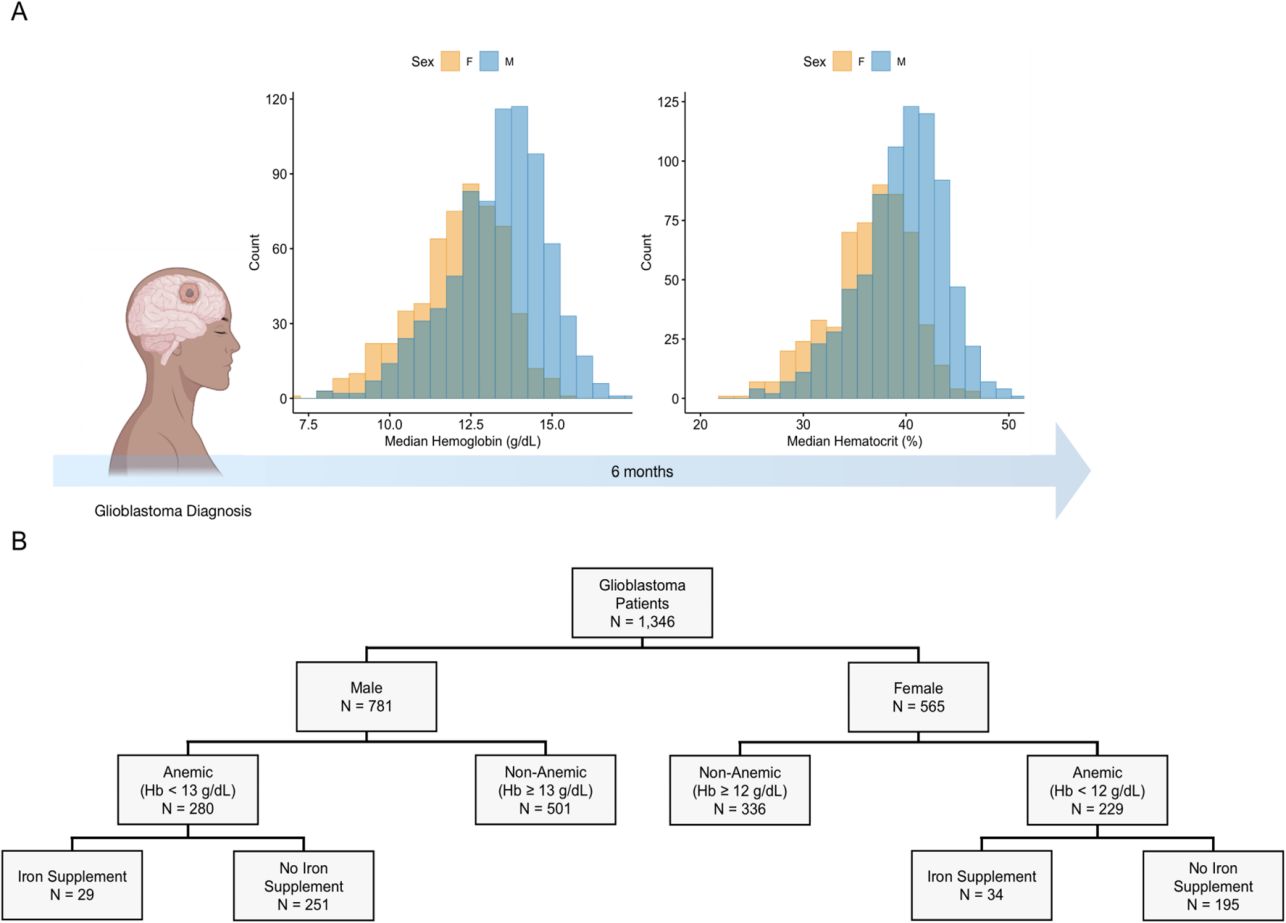
Anemia status and patient distribution. (**A**) Median hemoglobin and hematocrit levels within 6 months post glioblastoma diagnosis. (**B**) Distribution of the 1346 glioblastoma patients by sex, anemia status, and whether the anemic patients received an iron supplement. Created with help from biorender.com.

Among the 1346 glioblastoma patients in our cohort there were more males than females (781 vs. 565). Males had higher hemoglobin, (13.32 g/dL vs. 12.11 g/dL, *P* < 0.001), hematocrit (39.57% vs. 36.47%, *P* < 0.001), and white blood cell counts (9.58 × 10^9^/L vs. 9.03 × 10^9^/L, *P* = 0.024) compared to females (Fig. 1A and Supplementary Table 5). Males were also more likely to have smoked (50.6% vs. 36.8%, *P* < 0.001) or have abused alcohol (9.9% vs. 3.5%, *P* < 0.001) compared to females. Females were more likely to have had rheumatic diseases (3.5% vs. 0.8%, *P* < 0.001) compared to males (Supplementary Table 5).

Among the 565 females, 229 (40.5%) met the criteria for anemia using WHO guidelines of 12 g/dL for females. Among the 781 males, 280 (35.9%) met the criteria for anemia using WHO guidelines of 13 g/dL for males. Of the 229 anemic females, 34 (14.8%) were found to have received an iron supplement within 5 years prior or 6 months after glioblastoma diagnosis and 29 (10.4%) of the 280 anemic males were found to have received an iron supplement (Fig. 1B).

### Anemia in male patients is associated with reduced median overall survival

**Figure 2:**
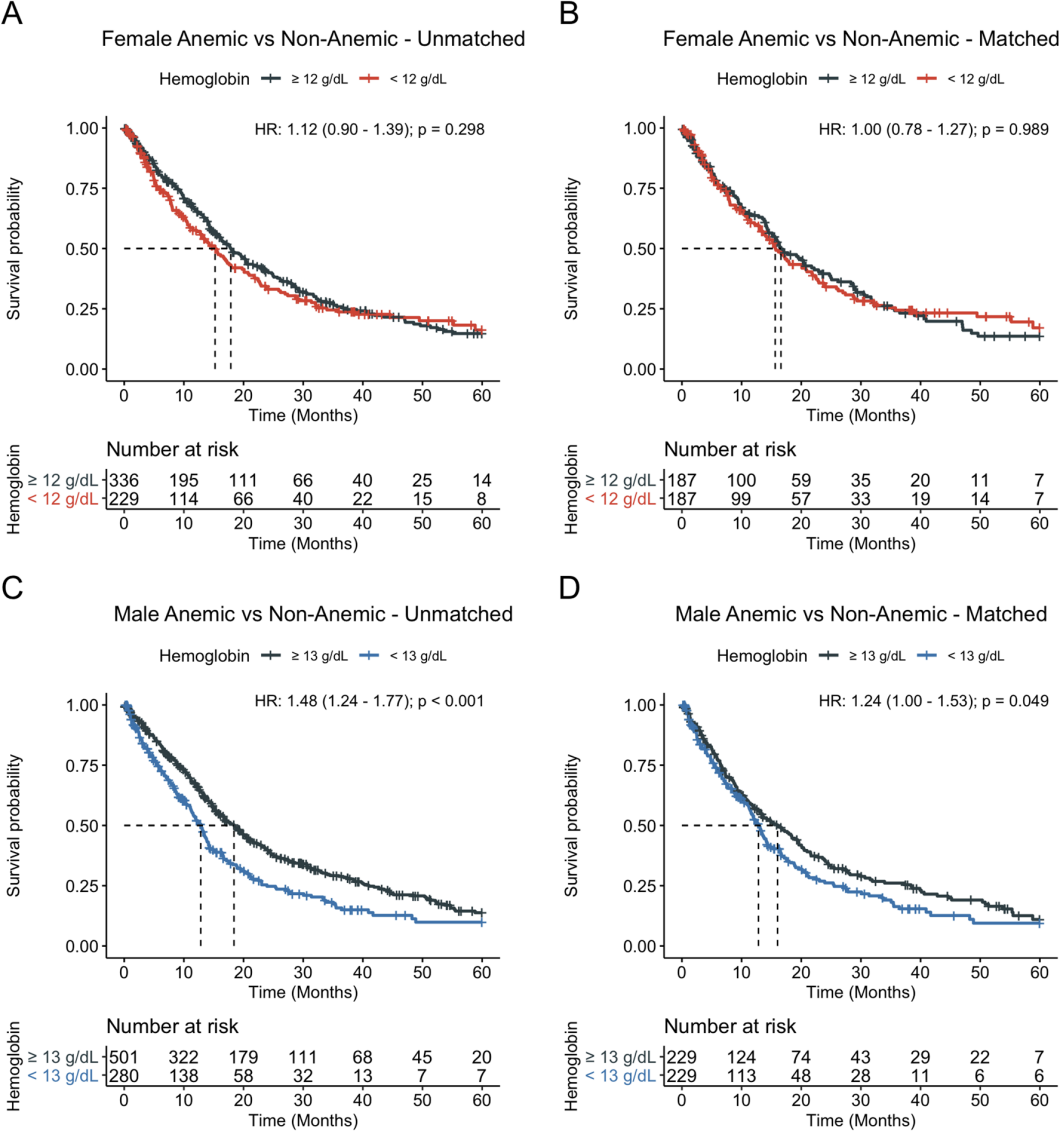
Sex-specific overall survival of patients stratified by anemic versus non-anemic using hemoglobin cutoffs. Sex-specific median overall survival was assessed for anemic versus non-anemic patients before (**A**, **C**) and after (**B**, **D**) propensity score matching. Anemia, as defined using World Health Organization cutoffs of < 12 g/dL for females and < 13 g/dL for males, was associated with reduced 5-year overall survival.

The association of anemia with median overall survival (m-OS) was assessed after classifying patients as anemic using WHO based hemoglobin cutoffs of 12 g/dL for females and 13 g/dL for males. To account for baseline differences between the anemic and non-anemic patients, the two cohorts were balanced using propensity score matching. After matching, balance between anemic and non-anemic cohorts was assessed for age at diagnosis, Charlson comorbidity index, white blood cell counts, total number of hemoglobin measurements, whether patients received chemotherapy and/or radiation, time interval between diagnosis to initiation of chemotherapy and/or radiation, and systemic comorbidities. The cohorts were found to be acceptably balanced using the commonly accepted threshold of 0.1 absolute mean standardized difference between covariates (Supplementary Fig. 1A,B) or by statistical testing (Tables 1, 2, Supplementary Tables 1, 2). Survival between balanced anemic and non-anemic cohorts stratified by sex was then assessed using Kaplan–Meier curves, log-rank testing, and univariate Cox regression with robust variance estimation. No association was found between anemia and median-OS in females (median-OS 15.6 vs. 16.6 months, *P* = 0.989, HR 1.00; 95% CI 0.78–1.27) (Fig. 2A,B) but anemia was found to be associated with reduced median-OS in males (median-OS 12.8 vs. 16.0 months, *P* = 0.049, HR 1.24; 95% CI 1.00–1.53) (Fig. 2C,D).

**Figure 3:**
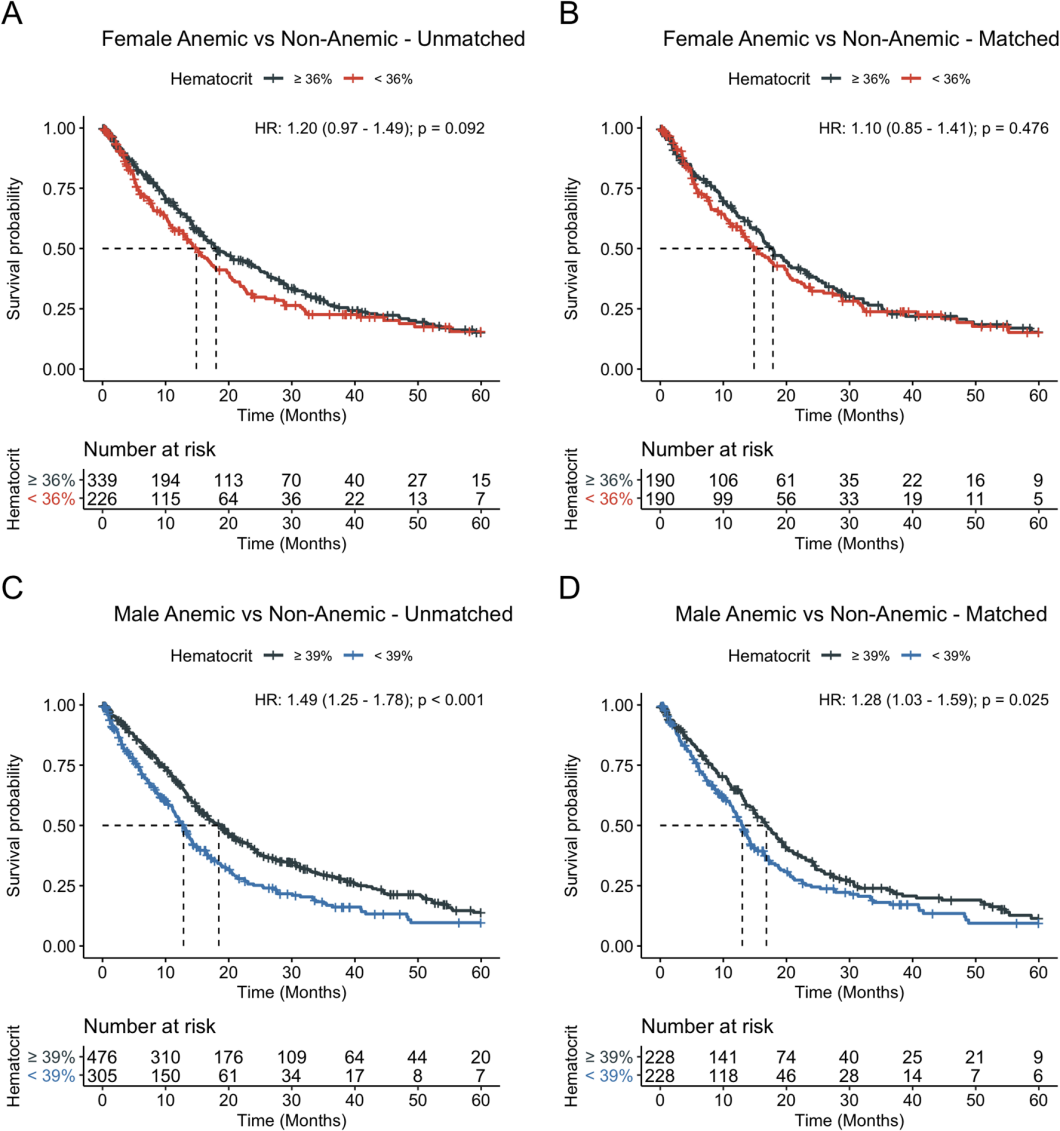
Sex-specific overall survival of patients stratified by anemic versus non-anemic using hematocrit cutoffs. Sex-specific median overall survival was assessed for anemic versus non-anemic patients before (**A**, **C**) and after (**B**, **D**) propensity score matching. Anemia, as defined using hematocrit cutoffs of < 36% for females and < 39% for males, was associated with reduced 5-year overall survival.

As an alternate to hemoglobin-based guidelines of anemia, hematocrit was used as the parameter for anemia classification. A hematocrit of 39% was defined as the anemia cutoff for males and 36% for females. After balancing cohorts as described above, balance was assessed and verified to be acceptable for all covariates apart from smoking status and rheumatic disease in males which had SMD slightly greater than 0.1 but acceptable balance on statistical testing (Supplementary Fig. 1C,D and Supplementary Tables 1 and 2). Sex-stratified Kaplan–Meier analysis, log-rank testing, and univariate Cox regression with robust variance estimation again revealed no association of anemia and median-OS in females patients (median survival 14.9 vs. 17.9 months, *P* = 0.476, HR 1.10; 95% CI 0.85–1.41) (Fig. 3A,B) but a strong association of anemia and reduced median-OS in males (median-OS 13.0 vs. 16.8 months, *P* = 0.025, HR 1.28; 95% CI 1.03–1.59) (Fig. 3C,D).

### Iron supplementation is associated with a trend toward improved survival in anemic female patients

**Figure 4:**
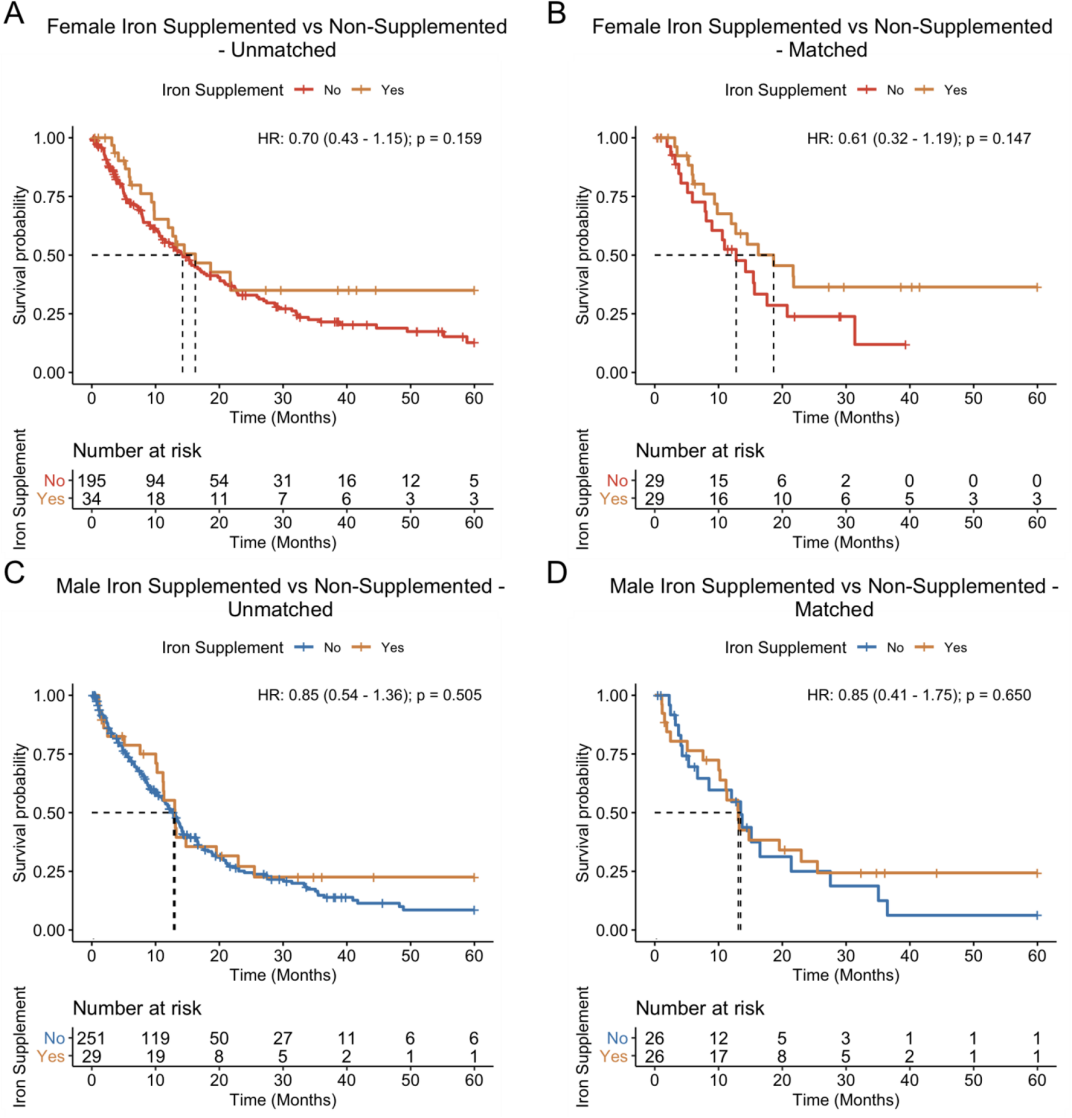
Overall survival of patients stratified by iron supplementation status and sex. Median overall survival was assessed for patients that received an iron supplement versus those that did not receive an iron supplement stratified by sex before (**A**, **C**) and after (**B**, **D**) propensity score matching. Iron supplementation was associated with prolonged overall survival in female patients but not in male patients.

We next investigated whether iron supplementation of anemic patients may be associated with survival. Among 280 anemic males, 29 (10.4%) were found to have received an iron supplement. Among the 229 anemic females, 34 (14.8%) received an iron supplement. The iron supplemented and non-supplemented patient cohorts were then stratified by sex and balanced for age at diagnosis, Charlson score, and whether patients received chemotherapy and radiation using propensity score matching to reduce bias. Because of limitations in sample size, balance was assessed only for age at diagnosis, Charlson comorbidity index, and whether patients received chemotherapy and/or radiation (Supplementary Fig. 1E,F). While there is insufficient sample size in the number of iron supplemented patients to achieve balance on every individual comorbidity, balance was achieved in the Charlson comorbidity index which serves as a general measure of baseline health of the patient cohort^35^. Performing sex-stratified Kaplan–Meier analysis and univariate Cox regression with robust variance estimation on the matched cohorts revealed that iron supplementation was associated with a trend toward prolonged survival in females although failing to reach statistical significance (median-OS 18.6 vs. 12.8 months, *P* = 0.147, HR 0.61; 95% CI 0.32–1.19) whereas no significant association was found in males after matching (median-OS 13.1 vs. 13.5 months, *P* = 0.650, HR 0.85; 95% CI 0.41–1.75) (Fig. 4).

### Male glioblastoma tumors exhibit increased transferrin binding

**Figure 5:**
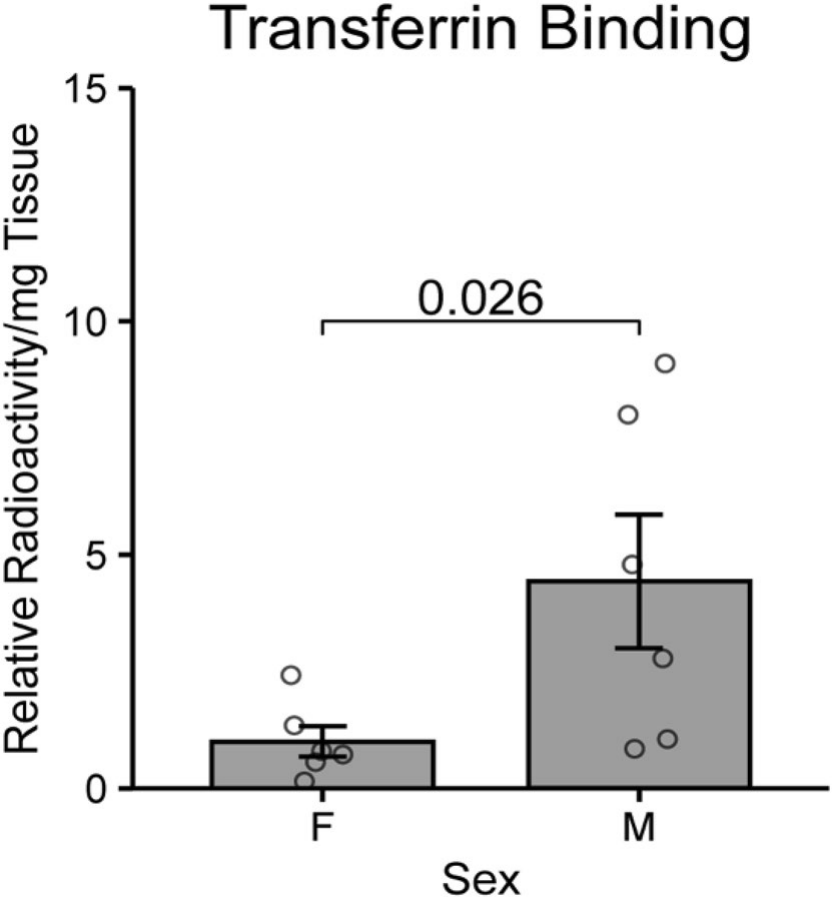
Transferrin binding in human glioblastoma samples by sex. Binding of ^125^I-tagged transferrin to extracted plasma membrane fractions from homogenized human glioblastoma tumors (n = 6 male, n = 6 female) was assessed by sex. Male tumors had significantly higher binding of radiolabeled transferrin compared to female tumors. P-value corresponds to unpaired two-sample Wilcoxon rank sum test.

Since sexual dimorphism was observed for both our analysis on anemia and iron supplementation, we hypothesized that there may be intrinsic differences in transferrin binding capacity between male and female tumors. To assess this, radiolabeled transferrin binding assays were performed on resected patient tumors. Since transferrin binding is tightly regulated by iron content, assessment of transferrin binding capacity of tissues represents a functional measure of tissue iron content^36,37^. Briefly, plasma membrane fractions of resected human glioblastoma samples were isolated and incubated with ^125^I-labeled transferrin. We found that plasma membrane fractions from male tumors bound significantly more transferrin per milligram of tissue compared to female tumor samples (Fig. 5). Of note, given that transferrin receptor is also highly expressed on endothelial cells, we cannot rule out that the male tumors may have had increased vascularization compared to the female tumors, however this would also suggest a mechanism where male tumors may have access to more iron^38^.

## Discussion

Anemia is a highly prevalent condition that is commonly found in cancer patients and often exacerbated by cancer treatment^2,3^. The role of anemia in glioblastoma patient outcomes is unclear with previous analyses being limited in number and reporting conflicting results. Some studies have found no association between anemia and survival^39^, while others have found a negative association of anemia with survival among glioblastoma patients^40–43^. These studies are summarized in Table 3 and reviewed in greater detail elsewhere^44^. Of note, these studies have been limited to single institutions and smaller patient cohorts with insufficient size to study sex as a biological variable. Here we report the largest, multi-center, analysis into the association of anemia and survival among adult glioblastoma patients to date. In our analysis of 1346 glioblastoma patients, we found that anemia within the 6 months following diagnosis was associated with reduced survival in male but not in female glioblastoma patients. We further investigated associations of iron supplementation with outcomes among anemic glioblastoma patients and found that supplementation of anemic female patients was associated with a trend toward prolonged survival although failing to reach significance, while no such association was found among anemic male patients.

**Table 3:** Summary of previous studies examining anemia and glioblastoma with references, year of publication, number of patients in study, and conclusion.

References	Year of publication	Total number of patients	Conclusion
Lutterbach et al.^39^	1999	N = 149	Anemia not associated with outcomes; RR: 0.91 (0.76–1.09), *P* = 0.32
Lutterbach et al.^40^	2003	N = 318	Anemia associated with worse outcomes; HR: 1.47 (1.13–1.90), *P* = 0.004
Odrazka et al.^41^	2003	N = 85	Anemia associated with worse outcomes; RR: 2.17 (1.33–3.54), *P* = 0.002
Ausili et al.^42^	2011	N = 43	Anemia associated with worse outcomes; HR: 2.44 (1.19–5.00), *P* = 0.010
Kaisman-Elbaz et al.^43^	2020	N = 112	Anemia associated with worse outcomes; HR: 1.79 (1.06–2.99), *P* = 0.031

The sexually dimorphic association of anemia with survival in our study raises interesting questions regarding the interplay of sex and tumor biology. Sexual dimorphism in glioblastoma has been well documented with males typically having higher incidence and poorer survival compared to females^1^. The analysis of transferrin binding to male and female tumors identified a potential underlying process contributing to the sexually dimorphic associations of anemia and iron supplementation with survival. Assessment of transferrin-binding capacity represents a functional measure of iron content in tumor tissues. When iron levels are low, transferrin receptor expression is elevated and vice-versa^36,37^. We found that membrane fractions from male tumors bound significantly more transferrin which is consistent with decreased iron stores in male tumors. The elevated transferrin binding in male tumors may represent an attempt by the tumor to increase iron acquisition due to decreased iron stores and is consistent with our observation that anemia seems to have a larger effect on male outcomes compared to female outcomes.

We also performed an analysis into associations between iron supplementation of anemic patients and median overall survival. After balancing iron supplemented and non-supplemented cohorts for age at diagnosis, Charlson Comorbidity Index, and whether chemotherapy and radiation were received, we found that iron supplementation in the period surrounding or after glioblastoma diagnosis was associated with a trend toward prolonged survival in females but not in males. Previous studies have suggested that iron supplementation, especially in combination with erythropoietin-stimulating agents, may be an effective approach at managing both absolute iron deficiency anemia as well as chemotherapy-induced functional anemia, but these studies did not investigate sex-differences in the context of glioblastoma^45,46^. Underlying differences in the etiology of the anemia between the sexes in our cohort may also contribute to the sexually dimorphic trends observed in the analysis on iron supplementation. Supporting this theory, we note that in our hemoglobin-stratified analysis, anemic female patients had a lower MCV than non-anemic female patients (Table 1: 89.68 vs. 91.44; *P* = 0.002) whereas no significant difference in MCV was found in the comparison between anemic versus non-anemic males (Table 2: 90.47 vs. 91.06; *P* = 0.234), suggesting that iron deficiency may contribute to a larger portion of the anemia observed in the female cohort compared to the male cohort and thus be more amenable to iron supplementation.

Many iron-related disorders including hereditary hemochromatosis and iron deficiency anemia exhibit marked sexual dimorphism in terms of incidence, age of onset, and severity of disease^47,48^. Females are more likely to be anemic compared to males due to physiological blood loss throughout much of adulthood and thus compensatory mechanisms may have evolved due to evolutionary adaptation resulting in sexually dimorphic handling of the downstream effects of anemia, including hypoxic responses^47^. Indeed, studies in animal models examining perinatal hypoxia-induced brain damage have reported that the hypoxia-induced injury was greater in males than in females^49,50^. Pathways activated by hypoxic responses such as those involved in cell death, handling of oxidative stress, and microglial activation have also been reported to exhibit features of sexual dimorphism^51^.

In addition to correcting anemia-induced hypoxic responses, iron itself may potentially contribute to anti-tumor responses. A study by Anic et al. examined iron content in toenail clippings collected from 193 glioblastoma patients post-diagnosis^52^. While that study did not examine associations between iron content and survival, it did report that toe nail iron appeared to decrease the further it was collected from the date of glioblastoma diagnosis, suggesting that glioblastoma pathology and iron biology may be more closely associated than previously appreciated^52^. Recent work has also demonstrated novel anti-tumor properties of iron including promoting ferroptosis^22^, inhibiting glioblastoma cell migration^23^, and promoting anti-tumor immunity^24^. In addition to differences in the underlying etiology of anemia, the sexual dimorphism observed in trends between iron supplements and survival may, in part, be explained by differences in iron uptake. Previous work in our laboratory demonstrated that, in mouse models, injection of radioactive iron bound to transferrin resulted in significantly higher uptake in female liver and brain compared to male controls suggesting sexually dimorphic regulation of body iron storage and blood–brain-barrier iron transport^53^.

Our study is limited by its retrospective nature and limited sample size for the analysis of iron supplementation of anemic patients. Iron supplements were infrequently utilized in the anemic glioblastoma patient cohort examined here and prospective clinical trials are needed to verify our findings. As these data were largely derived from de-identified electronic health records, data regarding molecular profiling is largely unavailable. Thus we cannot comment on whether differences in molecular profiling may have played a role our findings. In addition, our study lacks an independent validation cohort, yet does include a large sample size as a first investigation to be validated in a future study. Nonetheless, our study brings to attention a potentially clinically significant link between anemia and glioblastoma sex-specific outcomes that should be further investigated with anemia correcting therapies for use among glioblastoma patients to potentially both improve quality of life and provide more favorable outcomes.

### Supplementary Information

Supplementary Information.

## Data Availability

All de-identified unprocessed data is available on the TriNetX Research Network (https://trinetx.com). Processed aggregate data are provided in the supplementary information. Data from the transferrin binding assay is available upon request to the corresponding author.
