# Supplementary material for: Analysis of anemia and iron supplementation among glioblastoma patients reveals sex-biased association between anemia and survival
Source: Sci Rep. 2024 Jan 29;14:2389. doi: 10.1038/s41598-024-52492-8 (PMC10825121; doi:10.1038/s41598-024-52492-8)
Supplement: Supplementary file 1 — Supplementary Information. [file 41598_2024_52492_MOESM1_ESM.docx]

**Supplemental Data**

**Supplementary Figure 1.** Absolute standardized mean differences of covariates before and after propensity score matching.

**Supplementary Table 1.** Patient characteristics of anemic vs. non-anemic female glioblastoma patients using hematocrit cutoffs before and after matching.

**Supplementary Table 2.** Patient characteristics of anemic vs. non-anemic male glioblastoma patients using hematocrit cutoffs before and after matching.

**Supplementary Table 3.** Patient characteristics of iron supplemented vs. non-iron-supplemented anemic female glioblastoma patients before and after matching.

**Supplementary Table 4.** Patient characteristics of iron supplemented vs. non-iron-supplemented anemic male glioblastoma patients before and after matching.

**Supplementary Table 5.** Patient characteristics of overall cohort stratified by sex.

**Supplementary Table 6.** List of ICD-9 and ICD-10 codes used to calculate frequency of comorbidities and the Charlson Comorbidity Index.

**Supplementary Table 7.** List of RxNorm code(s) used to identify prescription of pharmaceuticals.

**Supplementary Table 8.** List of LOINC or TNX code(s) used to identify lab values

**
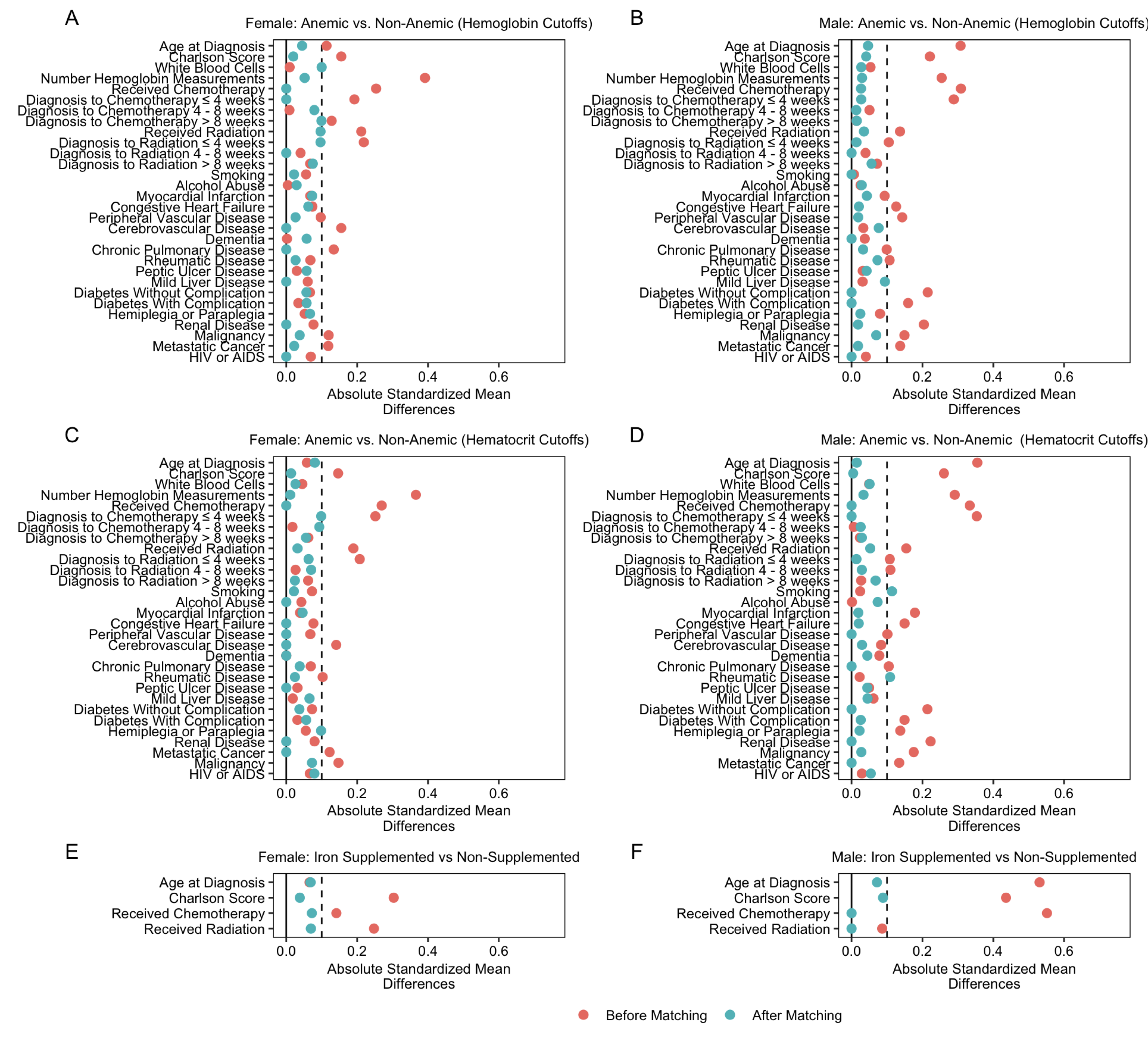
**

**Supplementary Figure 1. Absolute standardized mean differences of covariates before and after propensity score matching.** Balance was assessed between covariates before and after propensity score matching by calculating absolute standardized mean differences between treatment and control groups. A threshold of ≤ 0.1 absolute standardized mean difference was used to classify a covariate as balanced.

**Supplementary Table 1.** Patient characteristics of anemic vs. non-anemic female glioblastoma patients using hematocrit cutoffs before and after matching.

**Supplementary Table 2.** Patient characteristics of anemic vs. non-anemic male glioblastoma patients using hematocrit cutoffs before and after matching

**Supplementary Table 3.** Patient characteristics of iron supplemented vs. non-iron-supplemented female glioblastoma patients before and after matching.

**Supplementary Table 4.** Patient characteristics of iron supplemented vs. non-iron-supplemented male glioblastoma patients before and after matching.

**Supplementary Table 5.** Characteristics of overall cohort stratified by sex.

**Supplementary Table 6.** List of ICD-9 and ICD-10 codes used to calculate frequency of comorbidities and the Charlson Comorbidity Index.

| **Comorbidity** | **ICD-9 codes** | **ICD-10 codes** |
| --- | --- | --- |
| Myocardial infarction | 410, 412 | I21-I22, I25.2 |
| Congestive heart failure | 398.91, 402.01, 402.11, 402.91, 404.01, 404.03, 404.11, 404.13, 404.91, 404.93, 425.4-425.9, 428 | I09.9, I11.0, I13.0, I13.2, I25.5, I42.0, I42.5-I42.9, I43, I50, P29.0 |
| Peripheral vascular disease | 093.0, 437.3, 440-441, 443.1-443.9, 447.1, 557.1, 557.9, V43.4 | I70-I71, I73.1, I73.8, I73.9, I77.1, I79.0, I79.2, K55.1, K55.8, K55.9, Z95.8, Z95.9 |
| Cerebrovascular disease | 362.34, 430-438 | G45-G46, H34.0, I60-I69 |
| Dementia | 290, 294.1, 331.2 | F00-F03, F05.1, G30, G31.1 |
| Chronic pulmonary disease | 416.8, 416.9, 490-505, 506.4, 508.1, 508.8 | I27.8-I27.9, J40-J47, J60-J67, J68.4, J70.1, J70.3 |
| Rheumatic disease | 446.5, 710.0-710.4, 714.0-714.2, 714.8, 725 | M05-M06, M31.5, M32-M34, M35.1, M35.3, M36.0 |
| Peptic ulcer disease | 531-534 | K25-K28 |
| Mild liver disease | 070.22-070.23, 070.32-070.33, 070.44, 070.54, 070.6, 070.9, 570-571, 573.3-573.4, 573.8-573.9, V42.7 | B18, K70.0-K70.3, K70.9, K71.3-K71.5, K71.7, K73-K74, K76.0, K76.2-K76.4, K76.8-K76.9, Z94.4 |
| Diabetes without complication | 250.0-250.3, 250.8, 250.9 | E10.0-E10.1, E10.6, E10.8-E11.1, E11.6, E11.8-E12.1, E12.6, E12.8-E13.1, E13.6, E13.8-E14.1, E14.6, E14.8-E14.9 |
| Diabetes with complication | 250.4-250.7 | E10.2-E10.5, E10.7, E11.2-E11.5, E11.7, E12.2-E12.5, E12.7, E13.2-E13.5, E13.7, E14.2-E14.5, E14.7 |
| Hemiplegia or paraplegia | 334.1, 342-344.6, 344.9 | G04.1, G11.4, G80.1, G80.2, G81-G83.4, G83.9 |
| Renal disease | 403.01, 403.11, 403.91, 404.02-404.03, 404.12-404.13, 404.92-404.93, 582-583.7, 585-586, 588.0, V42.0, V45.1, V56 | I12.0, I13.1, N03.2-N03.7, N05.2-N05.7, N18-N19, N25.0, Z49.0-Z49.2, Z94.0, Z99.2 |
| Malignancy | 140-172, 174-195.8, 200-208, 238.6 | C00-C26, C30-C34, C37-C41, C43, C45-C58, C60-C76, C81-C85, C88, C90-C97 |
| Moderate or severe liver disease | 456.0-456.2, 572.2-572.8 | I85.0, I85.9, I86.4, I98.2, K70.4, K71.1, K72.1, K72.9, K76.5-K76.7 |
| Metastatic cancer | 196-199 | C77-C80 |
| AIDS/HIV | 042-044 | B20-B22, B24 |

**Supplementary Table 7.** List of RxNorm code(s) used to identify prescription of pharmaceuticals.

| **Comorbidity** | **RxNorm Code(s)** |
| --- | --- |
| Temozolomide | 37776 |
| Carmustine | 2105 |
| Lomustine | 6466 |
| Iron Supplements | 105669, 1102188, 1433693 1594675, 1607976, 2274394 24909, 24941, 24942, 24947 261435, 262150, 284110, 473387, 5992 |

**Supplementary Table 8.** List of LOINC or TNX code(s) used to identify lab values

| **Comorbidity** | **RxNorm Code(s)** | **TNX Code(s)** |
| --- | --- | --- |
| Hemoglobin | 718-7 | 9014 |
| Hematocrit | 20570-8, 4544-3 | - |
| Mean Corpuscular Volume | 787-2 | 9011 |
| White Blood Cell Counts | 49498-9, 26464-8, 6690-2, 804-5 | 9015 |
